# Obtaining Rotational Stiffness of Wind Turbine Foundation from Acceleration and Wind Speed SCADA Data

**DOI:** 10.3390/s25154756

**Published:** 2025-08-01

**Authors:** Jiazhi Dai, Mario Rotea, Nasser Kehtarnavaz

**Affiliations:** 1 Department of Electrical and Computer Engineering, University of Texas at Dallas, Richardson, TX 75080, USA; jiazhi.dai@utdallas.edu; 2 Center for Wind Energy, University of Texas at Dallas, Richardson, TX 75080, USA; rotea@utdallas.edu; 3 Department of Mechanical Engineering, University of Texas at Dallas, Richardson, TX 75080, USA

**Keywords:** monitoring of wind turbine foundation, rotational stiffness of wind turbine foundation, detection of wind turbine foundation degradation

## Abstract

Monitoring the health of wind turbine foundations is essential for ensuring their operational safety. This paper presents a cost-effective approach to obtain rotational stiffness of wind turbine foundations by using only acceleration and wind speed data that are part of SCADA data, thus lowering the use of moment and tilt sensors that are currently being used for obtaining foundation stiffness. First, a convolutional neural network model is applied to map acceleration and wind speed data within a moving window to corresponding moment and tilt values. Rotational stiffness of the foundation is then estimated by fitting a line in the moment-tilt plane. The results obtained indicate that such a mapping model can provide stiffness values that are within 7% of ground truth stiffness values on average. Second, the developed mapping model is re-trained by using synthetic acceleration and wind speed data that are generated by an autoencoder generative AI network. The results obtained indicate that although the exact amount of stiffness drop cannot be determined, the drops themselves can be detected. This mapping model can be used not only to lower the cost associated with obtaining foundation rotational stiffness but also to sound an alarm when a foundation starts deteriorating.

## 1. Introduction

Wind energy is a major contributor to renewable energy in generating electricity. Currently wind energy’s contribution to global electricity supply stands at 7.8% [1]. As wind farms expand in number and size, the need for maintaining the structural integrity of wind turbine foundations grows. Foundation failures can result in severe operational disruptions, safety risks, and substantial financial losses [2,3]. It is thus essential to monitor the condition of wind turbine foundations.

As discussed in [4], rotational stiffness is a key indicator that is used to assess the health of a wind turbine foundation. This indicator reflects the structural resistance of a foundation to external loads and thus provides early signs of foundation degradation, such as soil softening or structural damage. The current practice for obtaining foundation rotational stiffness involves establishing the linear relationship between foundation bending moment and tilt, which are measured by strain gauges and a tiltmeter installed on the wind turbine tower, respectively. However, as noted in [5,6], installing strain gauges and a tiltmeter on the tower and conducting measurements are tedious and costly. Furthermore, the current approach does not allow continuous monitoring of foundation stiffness since the sensors are normally removed after taking measurements.

To lower the cost associated with measuring foundation rotational stiffness, this paper offers an alternative approach by using only SCADA (Supervisory Control and Data Acquisition) data captured from the standard monitoring system of a wind turbine. The features or signals of the SCADA data used here for obtaining stiffness are acceleration and wind speed. Prior studies in [7,8,9,10,11,12] have utilized acceleration and wind speed to reflect structural dynamics, and thus, these features are used here for obtaining foundation rotational stiffness. It is worth mentioning that although natural frequencies of accelerometer signals have been utilized to assess the structural health of a turbine, for example [13,14,15,16], their applicability to estimating foundation rotational stiffness has been limited due to operational fluctuations, noise, and the presence of multiple overlapping natural frequencies of different turbine components.

A data-driven deep learning method is considered in this paper to establish the nonlinear relationship between acceleration/wind speed and direct moment/tilt measurements towards estimating foundation rotational stiffness. The first objective of this work is to demonstrate that foundation stiffness can be estimated using only SCADA acceleration and wind speed data, thus reducing the dependency on the installation of moment and tilt sensors on the tower. Once trained, the mapping model can be used to estimate foundation stiffness using only SCADA data. This allows limiting moment and tilt measurements to just recalibration of the model, and thus reducing the frequency and cost associated with conducting moment and tilt measurements. The second objective of this work is to detect a drop or degradation in foundation stiffness by considering synthetic data that are generated via a generative AI network, since real data corresponding to foundation degradation are scarce and not normally available. The synthetic data generated by a generative AI network can be used to determine whether the developed mapping model can detect a drop in stiffness to provide early warning in foundation health monitoring.

This paper addresses two main questions: (1) Is it possible to derive or estimate foundation rotational stiffness from acceleration and wind speed SCADA data? (2) Is it possible to detect a drop in stiffness using acceleration and wind speed SCADA data? This paper shows that the answer to both questions is affirmative, noting that this work is the first time the above issues related to wind turbine foundations are addressed.

## 2. Dataset and Data Preprocessing

**Figure 1:**
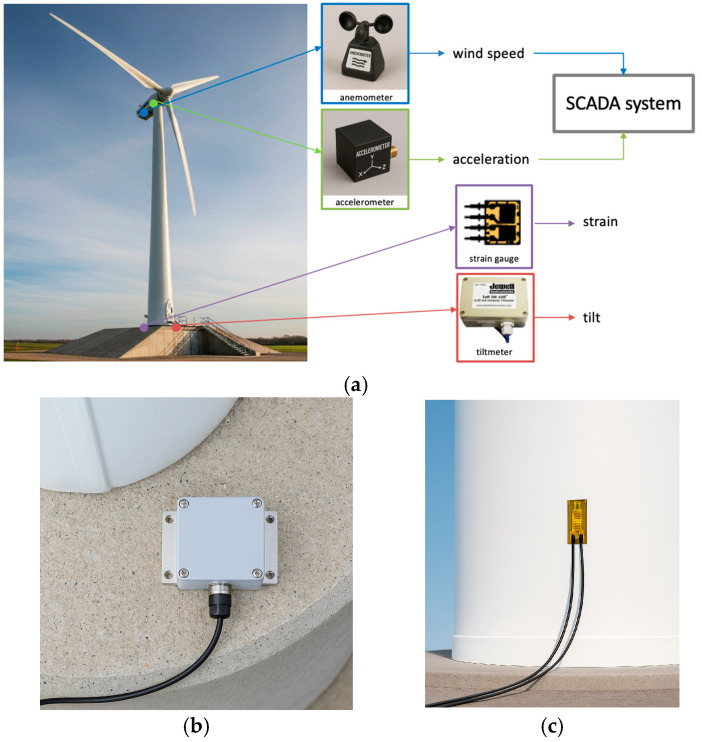
Illustrations of (**a**) the sensors and their locations for the data collection, (**b**) tiltmeter placed on foundation, (**c**) strain gauge placed on wind tower.

A dataset was provided for this work by a wind farm owner/operator company. The dataset consists of the SCADA data as well as the moment and tilt measurements for five turbines. Figure 1 provides illustrations of the sensors and their locations for the data collection.

**Figure 2:**
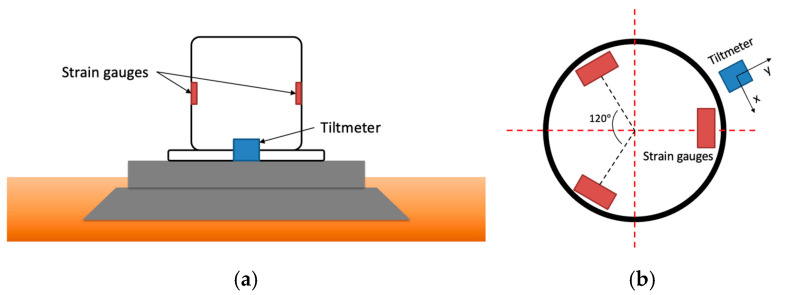
Locations of strain gauges and tiltmeter on a wind turbine tower: (**a**) side view and (**b**) top view.

The SCADA data includes the wind speed signal from a nacelle-mounted anemometer as well as the acceleration signal from an accelerometer installed on the nacelle. Tilt and moment were measured via a tiltmeter (Jewell Instrument C801 from Jewell Instruments, Manchester, NH, USA) at the base of the tower and three strain gauges (OMEGA KFH-3-120-D16-11L3M3S from Dwyer Instruments, Michigan City, IN, USA) installed around the tower spaced 120 degrees apart, see the illustration in Figure 2. For the specifications of these sensors, interested readers are referred to the manufacturers’ manuals [17,18].

Angle changes were recorded by the tilt sensor across two orthogonal axes, *x* and *y,* while the force-induced deformation of the tower/foundation was recorded by three strain gauges. In what follows, the computational details associated with these measurements are provided.

The dataset used in our work involves the resultant bending moment and the resultant tilt data. To estimate the stiffness of the wind turbine foundation, strain measurements are first converted into bending moments. According to Hooke’s Law, moment is given by [19]

(1) M=ϵ·E·S

where ϵ denotes strain, and E is the Young’s modulus of the tower material. For a hollow circular cross section, the section modulus *S* is given by [19]

(2) $$\;S=\frac{\pi \left(OD^{4}-ID^{4}\right)}{32\cdot OD}$$

where *OD* and *ID* denote the outer and inner diameters of the tower base, respectively. By combining the above expressions, the bending moment can be computed from the measured strain and the known tower geometry and material.

Since strain gauges are not always aligned with the thrust direction, the resultant bending moment is not obtained directly. To obtain the resultant bending moment, three strain gauges positioned in 120° intervals around the tower, as illustrated in Figure 1b, are used to generate the moments $M_{1},\;M_{2},\;M_{3}$, respectively. All the moments are combined into a resultant moment. The magnitude of the resultant moment from the strain gauges $m_{1},\;m_{2},or\;m_{3}$ is then obtained using the directional angle *α* between the resultant moment and the strain gauges as follows:

(3) $$\;m_{1}=\;\frac{M_{1}}{\cos\left(\alpha \right)}$$

(4) $$\;m_{2}=\frac{M_{2}}{\cos\left(\alpha +120^{\circ }\right)}$$

(5) $$\;m_{3}=\frac{M_{3}}{\cos\left(\alpha +240^{\circ }\right)}$$

Ideally, $m_{1}=\;m_{2}\;=m_{3}.\;$ However, in practice, to reduce measurement noise, the following average is used

(6) $$\;m=\frac{m_{1}+m_{2}+m_{3}}{3}$$

The tilt measurements are also integrated into a resultant tilt quantity *θ* according to the following equation

(7) $$\;\theta =\sqrt{{\theta_{x}}^{2}+{\theta_{y}}^{2}}$$

where $\theta_{x}$ and $\theta_{y}$ denote tilt values measured by the tiltmeter along *x* and *y* directions. In the dataset, the sampling rate of the SCADA data is at 1 Hz while the sampling rate of the tilt and moment data is at 10 Hz. The dataset covers a monitoring period of about 10 min for each turbine.

Rotational stiffness is defined as the slope of the line that is fitted to m vs.θ data samples via a first-order polynomial regression, that is

**Figure 3:**
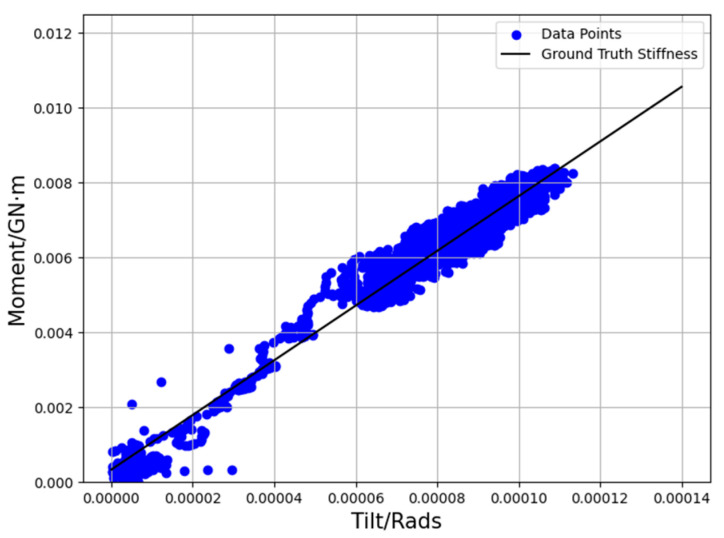
Moment and tilt sample points for a turbine (#24) in the dataset.

(8)  m=kθ+l

where the slope *k* is used to serve as the ground truth stiffness, with l being the *y*-intercept. Within a turbine’s normal operating condition, the relationship between tilt and moment is approximately linear, and the slope *k* reflects the structural integrity of the foundation. A linear regression is applied to the moment–tilt data pairs, and a goodness of fit based on the coefficient of determination *R*^2^ is computed, which varies between 0 and 1. A value close to 1 indicates there is a strong linear relationship between moment and tilt. Figure 3 shows the moment-tilt data pair samples and the best-fitted line or rotational stiffness for one of the turbines in the dataset.

For each turbine, the ground truth stiffness value (i.e., slope *k*), number of samples, and line goodness fit *R*^2^ are listed in Table 1. Although the turbines are of the same type, the variation in their stiffness values is due to the differences in the conditions of the foundations as well as their supporting soil. Despite variations in sample size and noise levels across the turbines, the tilt–moment relationship consistently follows a straight line.

Three data pre-processing steps were conducted on the dataset. First, since there was a 33 s misalignment in time between the moment/tilt data samples and the acceleration/wind speed data samples, a time shifting was performed to align the samples in time. Second, the acceleration and wind speed data samples were up-sampled to reach 10 Hz via linear interpolation in order to match the sampling rate of the moment and tilt data samples. Down-sampling to 1 Hz was not performed for several reasons: (i) Reducing the resolution of tilt and moment signals would have resulted in the loss of high-frequency information valuable for accurate stiffness estimation. (ii) Variations in the acceleration signal occur over short time durations and thus down-sampling would have suppressed them. Our analysis of the data indicated that when the window size exceeded 4 s, the measurement accuracy deteriorated. At 1 Hz sampling, the window would have included only three data points, which would have been insufficient for capturing signal variations. Third, FFT (Fast Fourier Transform) was applied to the acceleration signal to capture its frequency content within a window with the DC component removed. Wind speed was kept in the time domain to retain the temporal information. Several combinations of signal representations were examined, and it was found that using the frequency-domain acceleration along with the time-domain wind speed consistently yielded better stiffness estimation outcomes for the turbines examined. The above pre-processing steps were performed before using the mapping model discussed next.

## 3. Mapping Model

Given an input sequence $X\;=\;{\left\{\left(a_{t},\;v_{t}\right)\right\}}_{t=1}^{T}$, where $a_{t}$ indicates acceleration and $v_{t}$ wind speed at time *t*, a mapping model *f*(·) is designed to estimate tilt and moment outputs over a moving window in time with 50% overlap between consecutive windows, that is

(9) $$\;\left(\hat{\theta },\;\hat{m}\right)=f\left(X\right)$$

An estimated stiffness $\hat{k}$ is then obtained based on the estimated tilt and moment values by the mapping model as follows:

(10) $$\;\hat{k}=\frac{\sum_{i=1}^{N}\left({\hat{\theta }}_{i}-\bar{\hat{\theta }}\right)\left({\hat{m}}_{i}-\bar{\hat{m}}\right)}{\sum_{i=1}^{N}{\left({\hat{\theta }}_{i}-\bar{\hat{\theta }}\right)}^{2}}$$

where *N* denotes the number of data samples, ${\hat{\theta }}_{i}$ and ${\hat{m}}_{i}$ represent the *i*th estimated tilt and moment, and $\bar{\hat{\theta }}$ and $\bar{\hat{m}}$ their averages.

**Figure 4:**
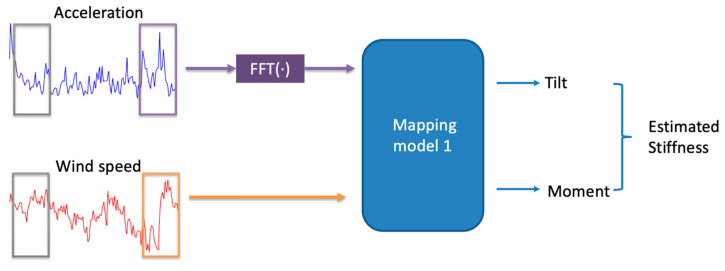
Mapping model.

The mapping model obtains a turbine’s foundation stiffness using only SCADA acceleration and wind speed data. As depicted in Figure 4, this mapping model (named model 1) is trained in a supervised manner for the purpose of estimating foundation stiffness from acceleration and wind speed signal inputs. The training set of a turbine is considered to be 70% of the dataset of that turbine, which is randomly selected, and the testing set is considered to be the remaining 30% of the dataset.

Four architectures were examined to conduct the mapping: CNN (Convolutional Neural Network), LSTM (Long Short-Term Memory), ANN (Artificial Neural Network), and Autoencoder. These models were selected based on their demonstrated success in time-series learning tasks in prior wind turbine studies [20,21,22,23]. Among these models, the CNN model performed the best, and thus the discussion that follows is focused on the architecture of this model.

### 3.1. Architecture of CNN Model

To estimate tilt and bending moment from the time-series input signals of acceleration and wind speed, a one-dimensional CNN is utilized as the mapping model. The input to the model is a sequence of windowed acceleration and wind speed values expressed by $X\;=\;{\left\{\left(a_{t},\;v_{t}\right)\right\}}_{t=1}^{T}\in R^{T\times 2}$, where *T* denotes the window size. The architecture of the CNN consists of the following layers:

(i) Input Layer—Accepts a sequence of 2-channel time-series/frequency map data over a window of size *T*.
(ii) First Convolutional Block—A 1D convolution layer with 32 filters of a kernel size 3. Then, ReLU (Rectified Linear Unit) activation is applied, followed by a max pooling layer that reduces the temporal dimension by a factor of 2

(11) $$\;H_{1}=MaxPool(\mathrm{ReLU}\left(X\;*\;W_{1}+b_{1}\right))$$

(iii) Second Convolutional Block—The feature map output of the first convolution block is passed through a second convolution layer with 64 filters (also with a kernel size 3, ReLU activation), followed by another max pooling layer

(12) $$\;H_{2}=MaxPool\left(\mathrm{ReLU}\left(H_{1}\;*\;W_{2}+b_{2}\right)\right)$$

(iv) Flatten and Dense Layers—The pooled features are flattened into a 1D vector and passed through a fully connected layer with 128 units and ReLU activation

(13) $$\;z=\mathrm{ReLU}\left(W_{3}\;*\;Flatten(H_{2})+b_{3}\right)$$

(v) Dropout Layer—To prevent overfitting, a dropout layer with a rate of 0.2 is added.
(vi) Output Layer—Finally, a dense layer with linear activation is used to output a 2D vector representing the estimated tilt and moment

(14) $$\;\left(\hat{\theta },\;\hat{m}\right)=W_{4}\;*\;z+b_{4}$$

The parameters of the model (*W*’s in the above equations) are obtained by minimizing the MSE (Mean Squared Error) loss function

(15) $$\;L_{MSE}=\frac{1}{N}\sum_{i=1}^{N}\left({\left({\hat{\theta }}_{i}-\theta_{i}\right)}^{2}+{\left({\hat{m}}_{i}-m_{i}\right)}^{2}\right)$$

**Figure 5:**
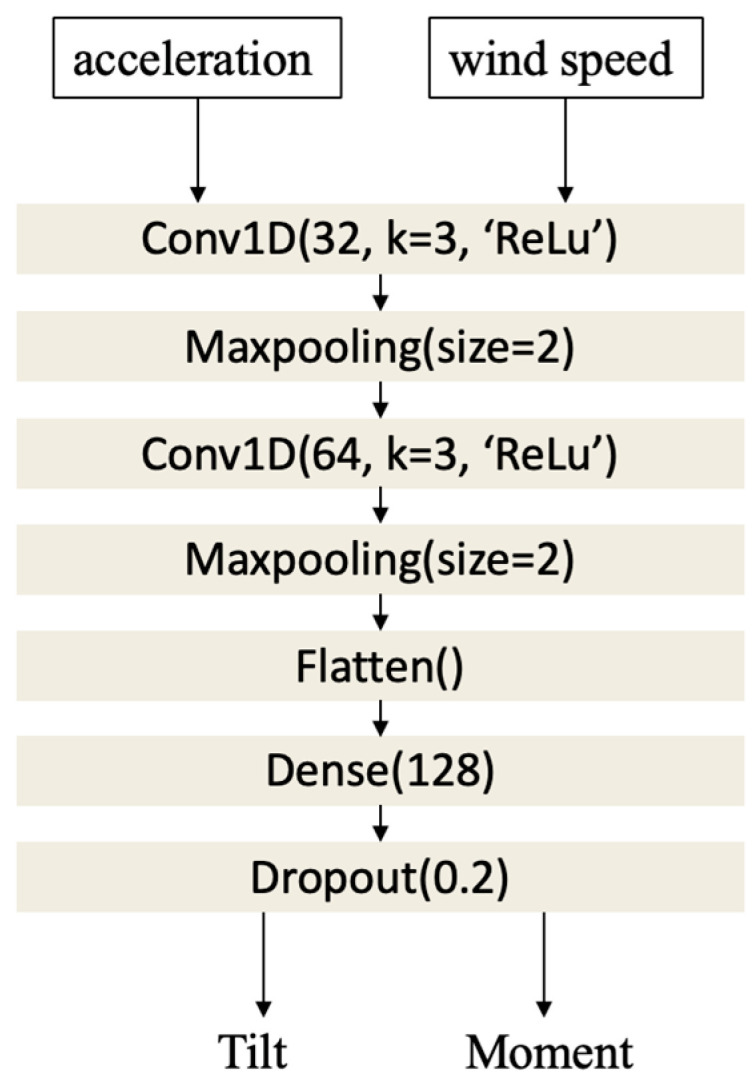
Architecture of the CNN mapping model used.

An illustration of the architecture considered is shown in Figure 5. The acceleration/wind speed input dimension of the CNN model is 30 (window size) + 30 (window size), and the tilt/moment output dimension of the CNN model is 2.

### 3.2. Window Size Selection

Careful selection of the sliding window size is needed for inputting data into the mapping model. A window that is too narrow may not adequately capture the hidden pattern in a signal due to the presence of noise, while a window that is too wide may obscure tilt and moment variations. An appropriate window size was determined empirically. Starting from a window size of 10 samples (corresponding to 1 s), the window size or length was incrementally increased by 10 samples. For each window size, 50 independent experiments were conducted, and the average error between the estimated and true tilt/moment values was computed. As shown in Table 2, a window size of 30 samples (3 s) was found to provide the best outcome in terms of generating the lowest mapping error.

## 4. Synthetic Data Generation

As stated in the introduction, besides the mapping of acceleration and wind speed to tilt and moment, it is desired to detect a drop in stiffness by the mapping model. However, no real SCADA data and tilt/moment measurements exist at lower stiffness values. The objective of detecting a drop in stiffness is met here by generating synthetic data based on real data. This section addresses the generation of such data.

Before the process of generating synthetic data is described, for detecting a drop in stiffness, the initial attempt comprised using the data from another turbine with lower stiffness. It was found that for two turbines with similar stiffness, the distributions or 2D histograms of moment (as well as tilt) vs. acceleration normalized by wind speed differed, indicating that the nonlinear relationship between acceleration/wind speed and tilt/moment also depends on the characteristics of a turbine. In other words, any synthetic data generated needs to come from the real data of the same turbine, not from the real data of a different turbine.

The generation of synthetic data is achieved here by using a generative AI network. The purpose of this network is to generate synthetic acceleration and wind speed signals corresponding to artificial tilt and moment data points at a lower stiffness or slope. A generative AI network is first trained by mapping real tilt/moment data samples to real acceleration/wind speed data samples, establishing the latent relationship between them. Then this network is used to generate synthetic acceleration/wind speed data samples from artificial tilt/moment data points at a lower stiffness. Artificial tilt/moment data points are obtained randomly along a line at a lower slope in the moment-tilt plane. After generating the synthetic data, the mapping network is retrained by using both the real and synthetic data.

**Figure 6:**
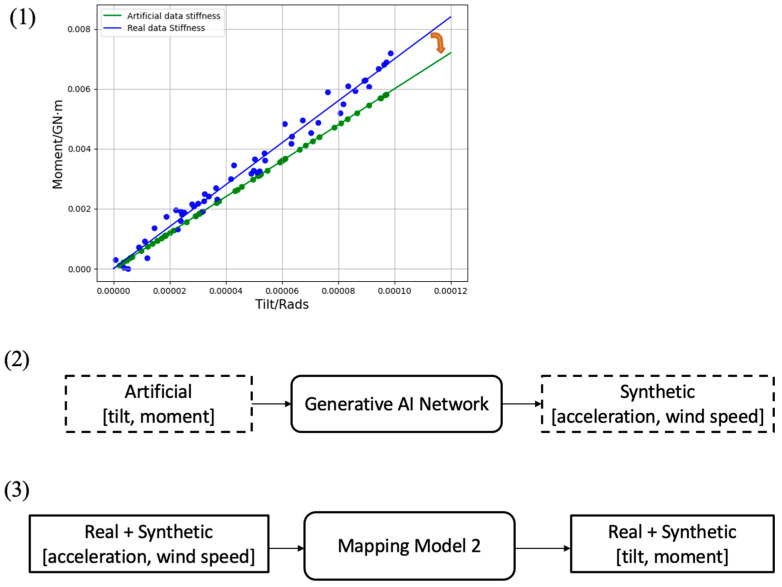
Steps taken to detect a drop in stiffness: (**1**) generating artificial moment and tilt data points, (**2**) generating synthetic acceleration and wind speed data samples from artificial moment and tilt data points, and (**3**) re-training the mapping model based on both real and synthetic data.

The above process is illustrated in Figure 6. As shown in this figure, the first artificial moment/tilt data points are generated on a line at a lower slope in the moment-tilt plane. The orange arrow in this figure indicates a simulated drop in stiffness. Then, a generative AI network is used to generate synthetic acceleration and wind speed data samples. Finally, both the real and synthetic data samples are used to train the mapping model (named model 2). In the next section, it will be shown that a drop in stiffness can be detected by model 2.

**Figure 7:**
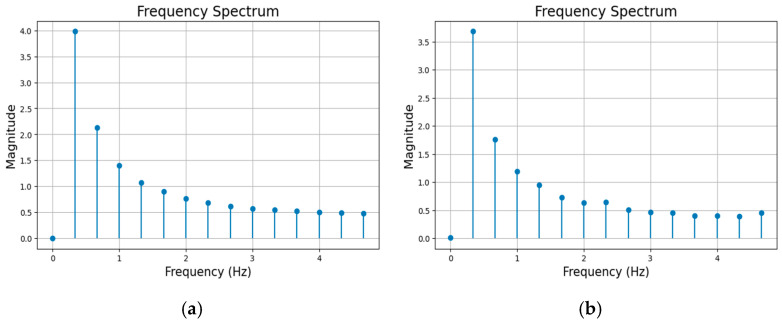
(**a**) Frequency spectrum of the real acceleration signal at the same wind speed, and (**b**) sample frequency spectrum of a synthetic acceleration signal generated by the AE network.

The generative AI networks of GAN (Generative Adversarial Network) [24], VAE (Variational Autoencoder) [25], and AutoEncoder (AE) [26] were examined for generating synthetic data. Among these generative AI networks, the AE network produced synthetic data that most closely resembled real data. Figure 7 provides an example illustrating the resemblance of the frequency spectrum of a sample synthetic acceleration signal generated by the AE network to the real acceleration signal at the same wind speed. Hence, in the subsection that follows, the focus is placed on the architecture of the AE network considered.

### Architecture of Autoencoder Network

An AE (AutoEncoder) network consists of an encoder and a decoder part. The encoder part takes a tilt-moment data pair and projects it into a low-dimensional latent representation space z.

(16) $$\;z=f_{encoder}\left(\theta ,m\right)$$

Then, the decoder reconstructs the corresponding acceleration and wind speed data from the latent representation.

(17) $$\;\hat{X}={\left\{\left(\hat{a_{t}},\hat{v_{t}}\right)\right\}}_{t=1}^{T}=f_{encoder}\left(z\right)$$

where $\hat{X}$ denotes the reconstructed acceleration $\hat{a_{t}}$ and wind speed $\hat{v_{t}}$.

**Figure 8:**
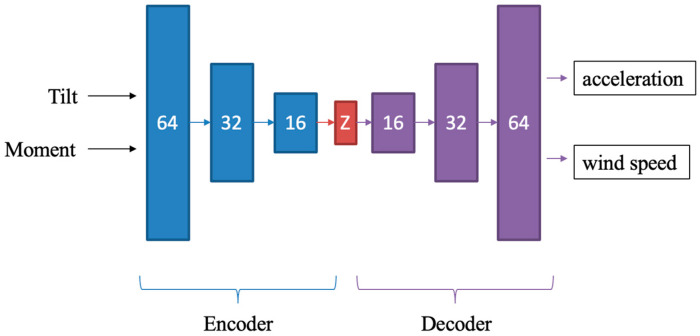
Architecture of the Autoencoder network used (numbers represent the number of so-called Dense units).

During training, the autoencoder is fed with the real tilt and moment data samples along with their corresponding real acceleration and wind speed data samples. The network learns to reconstruct the input by minimizing the reconstruction error between the original and generated sequences via minimizing the MSE loss function.

(18) $$\;L_{MSE}={\left\|X-\hat{X}\right\|}^{2}$$

where X denotes the original acceleration and wind speed signals. Once trained, the network is used to generate synthetic data. By inputting synthetic tilt and moment data samples from an artificially lower slope stiffness line, the network generates corresponding synthetic acceleration and wind speed data samples. The generated synthetic data samples allow the mapping model to detect a drop or reduction in stiffness. The architecture of the AE network used is displayed in Figure 8.

## 5. Results and Discussion

### 5.1. Results of Estimating Stiffness by the Mapping Model

For each turbine, the mapping model was trained 50 times, each time using a different training and testing set to remove any bias in the training/testing sets. The estimated tilt and moment values were then used to compute an estimated stiffness by fitting a linear regression line to the outputted tilt-moment pairs. The outcome of the mapping model is shown in Table 3. Basically, this table indicates that it is feasible to use acceleration and wind speed SCADA data to obtain a close estimate of the foundation rotational stiffness. The average difference between the estimated stiffness and the ground truth stiffness is found to vary from 1.8% to 7%, indicating the feasibility of using acceleration and wind speed for obtaining foundation rotational stiffness.

Noting that the cost associated with measuring tilt is relatively lower than that of measuring moment due to a more difficult installation of strain gauges on the tower, another experimentation was carried out by limiting the mapping model to only the moment. The outcome of this experimentation is shown in Table 4. As seen from this table, due to estimating only one quantity (moment), lower differences from the ground truth (0% to 5.3%) with narrower standard deviations were obtained as compared to Table 3 where two quantities (moment and tilt) were estimated.

### 5.2. Results of Detecting a Drop in Stiffness

First, to evaluate the similarity of synthetic data with real data, the cosine similarity metric was utilized. This metric captures the angular distance between two vectors representing the frequency components of synthetic acceleration signals with the frequency components of real acceleration signals. A similarity value close to 1 indicates a high similarity between real and synthetic data.

To establish a reliable baseline, the average cosine similarities between the real-to-real acceleration signals under the same wind speeds were first obtained. These values served as a reference for interpreting how realistic the generated synthetic signals were. Then, the average cosine similarities among the synthetic-to-real acceleration signals, also under the same wind speeds, were computed to evaluate how close the synthetic data came to replicating the real data.

**Figure 9:**
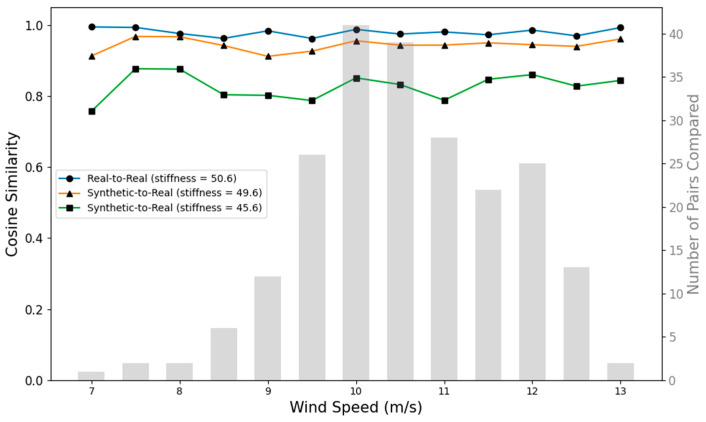
Average cosine similarities of real-to-real acceleration data (baseline) and average cosine similarities of synthetic-to-real acceleration data for 1 and 5 drops in stiffness (turbine #40).

More specifically, the original real acceleration dataset was randomly divided into two equal halves. The first half was used as the reference for comparison, ensuring it remained unseen by the generative process. The second half was used towards training the generative network and generating the synthetic data. The real-to-real (first half to second half) cosine similarities provided the baseline. The synthetic-to-real cosine similarities were then obtained for different levels of artificial drops in stiffness. Figure 9 shows the cosine similarity results for turbine #40. Similar results were obtained for the other turbines. As illustrated in this figure, the average synthetic-to-real cosine similarities were found to be close to the average real-to-real cosine similarities for artificial drops in stiffness below 5.

For each turbine, the mapping model was re-trained 50 times (different sets of training and testing sets) using 70% of both the real and synthetic data for training, which were randomly selected, and the remaining 30% of both the real and synthetic data were used for testing. The outcome of this mapping model (model 2) is shown in Table 5.

As seen from Table 5, although the detected drop in stiffness did not precisely match the artificial drop, they consistently reflected the drops in their values. Changes in soil properties, ground compaction, or other similar factors influence a turbine’s dynamic response, causing the mapping relationship to deviate from the artificial degradation scenarios examined. Nevertheless, what is of importance here is the fact that the utilization of the synthetic data allowed a drop in stiffness to be consistently detected.

## 6. Conclusions

A data-driven approach has been considered in this paper for the purpose of estimating the foundation rotational stiffness of wind turbines by using only acceleration and wind speed SCADA data. Such an approach can lower the cost associated with existing methods of measuring rotational stiffness, involving installing strain gauges and a tiltmeter on a turbine tower, by allowing less frequent utilization of these sensors or by reducing dependence on direct moment and tilt measurements. A convolutional neural network mapping model has been developed to map the acceleration and wind speed signals within a moving window to tilt and moment values. The training of the model is carried out on a per-turbine basis. It has been shown that this model provides estimated stiffness values that are close to the ground truth stiffness values. Furthermore, to detect a drop in stiffness for sounding an alarm, since no real data are available at lower stiffness values, synthetic acceleration and wind speed data have been generated by using an autoencoder generative AI network. As a result of including such synthetic data in the mapping model, it is shown that drops in stiffness can be detected. In summary, the developed approach provides a more cost-effective alternative solution to the existing approach of obtaining the rotational stiffness by installing moment and tilt sensors.

## Figures and Tables

**Table 1:** Ground truth stiffness, number of samples, and line goodness fit R^2^ of the dataset examined.

Turbine Number	Ground Truth Stiffness (GN·m/rads)	Number of Data Samples	R^2^
24	73.1	7200	0.95
37	66.5	2620	0.96
40	50.6	6610	0.81
44	41.2	6010	0.82
46	74.7	5780	0.93

**Table 2:** Window size selection.

Window Size (Samples)	Stiffness Estimation Absolute Error (GN·m/Rads)
10	4.5
20	2.7
30	1.3
40	4.2

**Table 3:** Stiffness estimation by the mapping model (model 1).

Turbine Number	Ground Truth Stiffness (GN·m/rads)	Estimated Stiffness (GN·m/rads) Mean ± Std	*t*-Distribution 95% Confidence Interval
24	73.1	74.4 ± 3.9	[73.2, 75.4]
37	66.5	69.9 ± 6.8	[67.9, 71.8]
40	50.6	53.2 ± 6.9	[51.3, 55.2]
44	41.2	44.1 ± 3.4	[43.2, 45.1]
46	74.7	77.2 ± 3.3	[76.3, 78.2]

**Table 4:** Stiffness estimation by the mapping model (model 1) with only moment as output.

Turbine Number	Ground Truth Stiffness (GN·m/rads)	Estimated Stiffness (Moment Only) (GN·m/rads) Mean ± Std	*t*-Distribution 95% Confidence Interval
24	73.1	73.4 ± 2	[72.8, 74.0]
37	66.5	66.5 ± 2.3	[65.9, 67.1]
40	50.6	51.4 ± 3.7	[50.3, 52.4]
44	41.2	43.4 ± 3.0	[42.6, 44.3]
46	74.7	77.1 ± 1.4	[76.7, 77.6]

**Table 5:** Results of detected drops in stiffness (model 2).

Turbine 24	Artificial drop in stiffness	−1	−2	−3	−4	−5
Measured stiffness	Estimated stiffness	72.6	72.8	72.5	72.5	72.0
73.1	Detected drop in stiffness	−0.1	−0.7	−1.0	−1.4	−1.6
Turbine 37	Artificial drop in stiffness	−1	−2	−3	−4	−5
Measured stiffness	Estimated stiffness	67.6	67.2	67.7	67.6	68.2
66.5	Detected drop in stiffness	−0.9	−1.3	−2.3	−3.0	−4.8
Turbine 40	Artificial drop in stiffness	−1	−2	−3	−4	−5
Measured stiffness	Estimated stiffness	50.7	50.3	50.9	50.6	50.0
50.6	Detected drop in stiffness	−0.1	−0.2	−0.9	−1.3	−1.6
Turbine 44	Artificial drop in stiffness	−1	−2	−3	−4	−5
Measured stiffness	Estimated stiffness	41.2	41.3	41.3	41.9	41.6
41.2	Detected drop in stiffness	−0.9	−1.6	−2.2	−3.6	−4.1
Turbine 46	Artificial drop in stiffness	−1	−2	−3	−4	−5
Measured stiffness	Estimated stiffness	74.8	74.8	74.5	74.3	74.3
74.7	Detected drop in stiffness	−0.4	−0.7	−0.8	−1.3	−1.6

## Data Availability

Dataset not available due to a nondisclosure agreement.
